# When Sleep Meets Stress: How Discrimination and Slow Wave Sleep Impact Memory in Aging Black Adults

**DOI:** 10.1093/geroni/igaf122.4046

**Published:** 2025-12-31

**Authors:** Osswaah Fariduddin, Stirling Crawford, Maria Clark, Elizabeth Luth, Darlingtina Esiaka

**Affiliations:** University of Kentucky, Lexington, Kentucky, United States; University of Kentucky, Frankfort, Kentucky, United States; University of Kentucky, Lexington, Kentucky, United States; Rutgers University, New Brunswick, New Jersey, United States; University of Kentucky - College of Medicine, Lexington, Kentucky, United States

## Abstract

**Background:**

Black Americans disproportionately experience everyday discrimination—unjust treatment based on race, limiting opportunities—which has been associated with risk of impaired visuospatial memory, an early indicator of cognitive decline. Slow wave sleep (SWS) is critical for memory consolidation but naturally declines with age. The decline is further exacerbated by psychosocial stressors such as discrimination. The purpose of this study is to examine whether SWS moderates the association between visuospatial memory and discrimination among aging Black Americans.

**Methods:**

As part of an ongoing study, participants (N = 59, Mage=52.76) completed three nights of at-home objective sleep monitoring using a wearable device (Sleep profiler) and measures assessing demographics and discrimination. Visuospatial memory was measured using the Benson Complex Figure Task. PROCESS Macro (Model 1) was used to test the moderating role of SWS on the association between discrimination and visuospatial memory, while adjusting for age, sex, and marital status.

**Results:**

SWS significantly moderated the relationship between discrimination and visuospatial memory (B=-0.0093, p = 0.047). At lower levels of discrimination, SWS had a positive association with visuospatial memory (B = 0.13, p = 0.089). However, at higher levels of discrimination, the association was negative (B=-0.089, p = 0.32). No significant main effects were found for age, sex, or marital status.

**Conclusion:**

Findings suggest that while SWS may be beneficial for cognitive health, the benefit is eroded by the presence of discrimination in aging Black Americans. Our study highlights how the combined effects of psychological and biological factors can provide crucial insight into cognitive aging among populations at high-risk of developing cognitive decline.

